# Characterization of Neutrophil Function in Human Cutaneous Leishmaniasis Caused by *Leishmania braziliensis*

**DOI:** 10.1371/journal.pntd.0004715

**Published:** 2016-05-11

**Authors:** Jacilara Conceição, Richard Davis, Pedro Paulo Carneiro, Angela Giudice, Aline C. Muniz, Mary E. Wilson, Edgar M. Carvalho, Olívia Bacellar

**Affiliations:** 1 Serviço de Imunologia, Hospital Universitário Professor Edgard Santos, Universidade Federal da Bahia, Salvador, Bahia, Brazil; 2 Interdisciplinary Program in Immunology, University of Iowa, Iowa City, Iowa, United States of America; 3 Department of Internal Medicine and Microbiology, University of Iowa, Iowa City, Iowa, United States of America; 4 Department of Veterans’ Affairs Medical Center, Iowa City, Iowa, United States of America; 5 Instituto Nacional de Ciência e Tecnologia de Doenças Tropicais - INCT-DT (CNPq/MCT), Salvador, Bahia, Brazil; 6 Centro de Pesquisa Gonçalo Moniz, FIOCRUZ – BA, Salvador, Bahia, Brazil; University of Texas Medical Branch, UNITED STATES

## Abstract

Infection with different *Leishmania* spp. protozoa can lead to a variety of clinical syndromes associated in many cases with inflammatory responses in the skin. Although macrophages harbor the majority of parasites throughout chronic infection, neutrophils are the first inflammatory cells to migrate to the site of infection. Whether neutrophils promote parasite clearance or exacerbate disease in murine models varies depending on the susceptible or resistant status of the host. Based on the hypothesis that neutrophils contribute to a systemic inflammatory state in humans with symptomatic *L*. *braziliensis* infection, we evaluated the phenotype of neutrophils from patients with cutaneous leishmaniasis (CL) during the course of *L*. *braziliensis* infection. After *in vitro* infection with *L*. *braziliensis*, CL patient neutrophils produced more reactive oxygen species (ROS) and higher levels of CXCL8 and CXCL9, chemokines associated with recruitment of neutrophils and Th1-type cells, than neutrophils from control healthy subjects (HS). Despite this, CL patient and HS neutrophils were equally capable of phagocytosis of *L*. *braziliensis*. There was no difference between the degree of activation of neutrophils from CL versus healthy subjects, assessed by CD66b and CD62L expression using flow cytometry. Of interest, these studies revealed that both parasite-infected and bystander neutrophils became activated during incubation with *L*. *braziliensis*. The enhanced ROS and chemokine production in neutrophils from CL patients reverted to baseline after treatment of disease. These data suggest that the circulating neutrophils during CL are not necessarily more microbicidal, but they have a more pro-inflammatory profile after parasite restimulation than neutrophils from healthy subjects.

## Author Summary

*Leishmania* spp. are protozoan parasites that cause a spectrum of human diseases, and *L*. *braziliensis* causes chronic inflammatory skin lesions in residents of endemic regions of Latin America. Leishmania are obligate intracellular parasites in mammalian hosts, found in macrophages throughout infection. Nonetheless, other cell types including neutrophils also take up the parasite, but the role of neutrophils throughout chronic leishmaniasis remains unclear. We analyzed circulating neutrophils from patients in northeast Brazil with cutaneous leishmaniasis (CL) caused by *L*. *braziliensis*, compared to healthy controls from the same region. Our data revealed that neutrophils from both infected and healthy hosts took up comparable numbers of parasites, and parasite phagocytosis induced similar degrees of neutrophil activation. However, CL patient neutrophils produced more reactive oxidants than control neutrophils, and increased amounts of the chemokines CXCL8 and CXCL9 after parasite exposure. Interestingly, according to surface markers of PMN activation (CD62L, CD66b), we found that *L*. *braziliensis* activates both infected and uninfected “bystander” neutrophils from both patients and controls. Importantly, repeated measures showed the production of reactive oxidants and chemokine release were significantly decreased after therapeutic cure of infection. These data suggest that CL promotes a heightened inflammatory state in circulating neutrophils during active infection.

## Introduction

Cutaneous leishmaniasis (CL) is the most common form of human leishmaniasis, a group of diseases caused by the *Leishmania* spp. protozoa. CL is widely distributed, but Brazil is among the countries with the highest estimated disease prevalence [1]. Within Latin America, *Leishmania braziliensis* is the most common cause of CL and other related tegumentary forms of leishmaniasis, including mucosal and disseminated leishmaniasis. Patients with CL due *L*. *braziliensis* develop a strong Th1-type adaptive immune response with high levels of IFN-γ and TNF-α produced primarily by CD4+ T cells [2–4]. These responses facilitate the control of parasite proliferation within by macrophages, but they also contribute to the pathologic changes that characterize disease [4–6].

The *Leishmania* spp. are obligate intracellular parasites in their mammalian hosts, and most are found within macrophages of infected tissues. In addition to macrophages, other cell types such as dendritic cells and neutrophils participate in the pathogenesis of *Leishmania* infection. Studies of mouse models show that neutrophils migrate to the site of infection soon after the sand fly bite, and are the first infiltrating cells to encounter *L*. *major* [7–9]. Migration of neutrophils to sites of infection is mediated by the interactions between endothelial cells and adhesion molecules expressed on neutrophil surfaces, which allow for binding and “rolling” prior to extravasation from vasculature [10]. Neutrophils may influence adaptive immune responses by producing chemokines, which recruit others cell types that in turn participate in the response to infection [11,12]. A partial list of neutrophil microbicidal responses includes assembly of the multi-protein NADPH oxidase complex with resultant production of reactive oxygen species, release of granule contents into intracellular microbial compartments, and release of defensins [13,14].

The role of neutrophils in *Leishmania spp*. infection has been predominantly studied in murine models, and findings have varied depending on both the species of *Leishmania* used and the resistance or susceptible genetic background of the mouse [15–17]. Confusing the picture, some methods for depletion of neutrophils in mice also deplete other critical cell subsets (e.g., dendritic cells, monocytes and macrophages), depending on the choice and dose of depleting antibody [18,19]. There is evidence that a subset of *L*. *donovani* survive intracellularly in murine neutrophils, raising the question whether neutrophils represent a “safe haven” facilitating parasite survival prior to delivery to its permanent host cell, the macrophage [20]. Neutrophils are also found to kill parasites, documented in experimental model of *L*. *braziliensis* infection, in which infection trigger neutrophil activation, increased ROS production and this leads to parasite clearance [21,22].

Studies of human neutrophils suggest there could also be an important role for these cells in human leishmaniasis. Neutrophils from healthy donors infected with *L*. *major* produce a strong oxidative response that eliminates internalized parasites [23]. Infection with *L*. *amazonensis* promotes neutrophils activation, degranulation and production of leukotriene B_4_ which promotes parasite killing [24]. Additionally, interactions between healthy human neutrophils and *Leishmania*-infected macrophages modulate the intracellular replication of both *L*. *amazonensis* [25] and *L*. *braziliensis* [26]. Based upon the hypothesis that neutrophils contribute to the inflammatory environment observed during infection, the current study was initiated to evaluate the phenotype of neutrophils from patients with CL due to *L*. *braziliensis* infection. Our data showed that neutrophils from CL patients and from healthy controls display both distinct and common characteristics. The phenotype of circulating neutrophils in CL subjects suggested that they behaved more like primed neutrophils, poised for rapid activation, in contrast to resting neutrophils from healthy subjects [27,28].

## Methods

### Ethics statement

All samples were obtained specifically for this study. The study was approved by Institutional Review Boards (IRBs) of the Federal University of Bahia (Ethical Committee), the University of Iowa and the NIH. Written informed consent was obtained from all participants. The UFBA IRB is registered with the NIH.

### Study area

Corte de Pedra is a village belonging to the municipality of Presidente Tancredo Neves, located in the southern region of the state of Bahia, Brazil. This endemic area is the most prevalent area for *L*. *braziliensis* transmission in Brazil, and more than a thousand cases of CL are seen in the Health Post in Corte de Pedra annually [29].

### Subjects

Participants in this study included 21 CL patients diagnosed at the Corte de Pedra Health Post. Diagnosis was based on the presence of clinical manifestations characteristic of CL, confirmed with at least one of three methods: parasite isolation, identification of amastigotes by histopathologic examination studies of biopsies, or a positive quantitative polymerase chain reaction test (qPCR) specific for parasite DNA derived from parasite tissue samples [30]. A control group was composed of 17 healthy Brazilian subjects (HS) who resided in a non-endemic area of northeast Brazil. As a part of their medical care, after confirmed diagnosis, patients underwent treatment with pentavalent antimony (Sb^v^), which is standard therapy for leishmaniasis in Brazil. CL patients received intravenous Sb^v^ at a dosage of 20 mg per kg of body weight per day over 20 days. Subjects were observed throughout the course of therapy and evaluated after completion of therapy for cure. Patients were considered cured of there was complete healing of lesions 90 days after initiation of therapy, with skin re-epithelialization and the absence of raised borders [31].

### Parasite culture and CFSE staining

A single *L*. *braziliensis* isolate (MHOM/BR/LTCP11245) was used in all experiments. This isolate was obtained from a skin lesion of a CL patient from Corte de Pedra and was characterized as *L*. *brazilensis* using both a qPCR assay and the standard isoenzyme electrophoretic mobility assay [32]. Parasites were cryopreserved after isolation in biphasic medium (NNN) after isolation. Before use, parasites were grown in Schneider’s medium (Aldrich Sigma, St. Louis, MO, USA) supplemented with 10% heat-inactivated fetal bovine serum (FBS) (Gibco-Life Technologies, Grand Island, NY, USA) and 2% sterile urine [33] obtained from healthy volunteers after informed consent.

Parasites were labeled with carboxyfluorescein succinimidyl ester (CFSE) (Invitrogen) as previously described [34]. Briefly *L*. *braziliensis* promastigotes were washed in saline and resuspended at 6x10^7^ in 10 ml of saline with 5 μM of CFSE, and incubated at 37°C for 5 minutes. Then parasites were washed twice saline containing FBS and resuspended in RPMI.

### Isolation of peripheral blood neutrophils

Neutrophils were obtained from heparinized venous blood by density gradient centrifugation using Ficoll Hypaque (LSM; Organon, Durham, NC, USA). The PBMC monolayer was collected from above the Ficoll layer, and erythrocytes were removed from the layer below Ficoll by Dextran sedimentation (Pharmacosmos A/S, Denmark) leaving a population of predominantly neutrophils [35]. The purity of neutrophils isolated using this technique was 95–99%, determined by microscopy using May-Gruenwald-Giemsa staining of cytocentrifuged slides. The cell concentration was adjusted to 1x10^6^/ml in complete culture media, consisting of RPMI 1640 (Gibco-Life Technologies, Grand Island, NY, USA) supplemented with 100U penicillin/ml, 100 μg streptomycin/ml and 10% heat-inactivated fetal bovine serum (FBS) or 10% autologous serum.

### *In vitro* infection with *Leishmania braziliensis*

One x 10^6^ neutrophils were co-incubated with *L*. *braziliensis* promastigotes at a parasite to PMN ratio of 5:1, 37°C, 5% CO_2_, in 1 ml of complete medium with 10% autologous serum. Neutrophils were stimulated with 10 ng/ml Phorbol 12-myristate 13-acetate (PMA) as a positive control.

After 10, 90 or 180 minutes of incubation, cytocentrifuge slides were prepared and stained with Giemsa, and the numbers of infected cells and intracellular *L*. *braziliensis* per 100 neutrophils were quantified by optical microscopy.

### Neutrophil cell surface staining and flow cytometric analysis

After incubation at 37°C, 5% CO_2_, cells were incubated at 4°C for 15 minutes, stained with fluorochrome conjugated monoclonal antibodies, and suspended in saline. Antibodies were: CD16 PE, CD62L-PECy7 and CD66b-PerCPCy5.5 (BD Pharmingen, San Diego, CA, USA). Flow cytometry data (at least 50,000 events per sample) were acquired using either a FACSVerse Flowcytometer (BD Bioscience) or a FACS CantoII (BD Bioscience). Data were analyzed using FlowJo software (Tree Star Inc., Ashland, OR, USA).

Neutrophils were identified by forward- and side-scatter characteristics and, in some cases, CD16+ expression. Infected or bystander population of neutrophils were identified as CFSE+ or CFSE- cells, respectively (S1 Fig).

As the experiments with healthy controls subjects were performed at the laboratory at Complexo Hospitalar Universitário Professor Edgard Santos in Salvador city, we used a different flow cytometry than we have at the laboratory in the endemic area.

### Reactive oxidants produced by neutrophils in response to *L*. *braziliensis* infection

The production of reactive oxygen species (ROS) was evaluated by flow cytometry using the fluorogenic substrate dihydrorhodamine 123 (DHR 123, Cayman Chemical Company, Ann Arbor, MI, USA) as an indicator. Briefly, 1x10^6^ neutrophils were incubated with 10 ng/ml of DHR123 after which either *L*. *braziliensis* promastigotes, PMA or buffer (control) was added. Control samples containing no stimulus were run in parallel. After 15 minutes incubation at 37°C, 5% CO_2_, cells were washed in PBS and analyzed by flow cytometry. The neutrophil population was gated on the basis of forward and side scatter followed by DHR123 fluorescence. Separate controls verified that this population corresponded to neutrophils according to CD16+ surface stain.

### Reactive oxidants inhibition and *in vitro* infection evaluation

In a separate experiment, neutrophils were treated with 10 mM of NADPH oxidase inhibitor, Diphenyleneiodonium chloride (DPI) prior to parasite exposure. The frequency of infected cells and the parasite burden were evaluated by microscopy.

### Parasite viability

Parasite viability was evaluated by enumeration after recovery in culture as previously described [24]. Briefly, after 180 minutes of infection with *L*. *braziliensis*, neutrophils were incubated in Schneider’s medium at 24°C for an additional 24 hours. *L*. *braziliensis* viability was measured by assessing the number of extracellular motile promastigotes.

### Chemokines measurement in neutrophil culture supernatants

After incubation with *L*. *braziliensis*, PMA or medium, neutrophil culture supernatants were collected. Chemokine levels (CCL4, CXCL9, CXCL8, CXCL10) were measured by sandwich ELISA according to the manufacturers’ instructions (R&D Systems, Minneapolis, MN, USA).

### Statistical analysis

A nonparametric Wilcoxon Signed-Rank Test was used to compare the results obtained with cells in different conditions from the same subject. A nonparametric Mann-Whitney U-test was used to evaluate differences among the groups. Statistical analyses were performed using GraphPad Prism 4.0 (GraphPad Software, Inc., San Diego, CA, USA). An alpha of P<0.05 was considered statistically significant.

## Results

### The ability of neutrophils from CL patients versus healthy subjects to phagocytose *L*. *braziliensis* is similar

**Fig 1:**
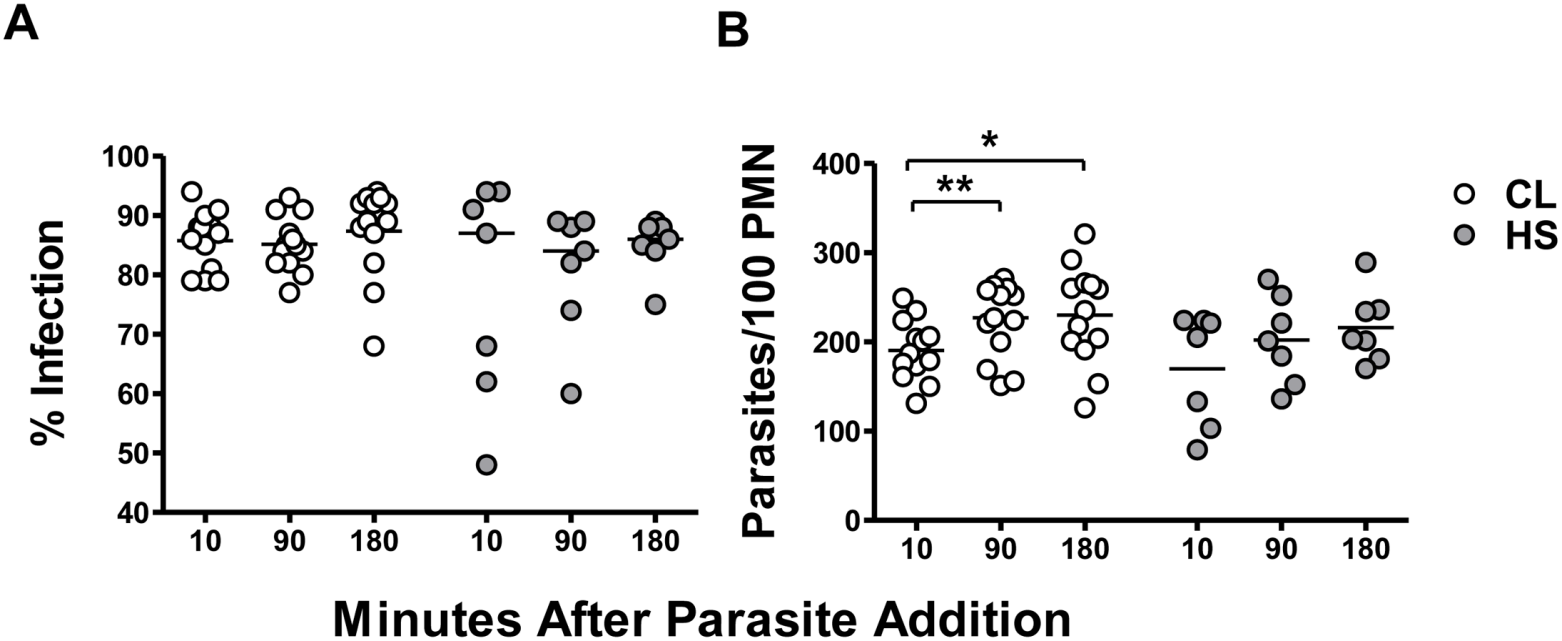
Uptake of *L*. *braziliensis* by neutrophils (PMN) from CL patients or healthy subjects (HS). Neutrophils from CL patients (n = 13) or healthy subjects (n = 7) were incubated with stationary phase *L*. *braziliensis* at a 5:1 parasite:neutrophil ratio, under conditions that allow phagocytosis. After 10, 90 or 180 minutes of incubation at 37°C, 5% CO_2_, cytocentrifuge slides were prepared and stained with Giemsa. The percentage of infected cells (A) and the number of intracellular *L*. *braziliensis* (B) per 100 neutrophils were determined microscopically. Each symbol represents the mean value of neutrophils from a different patient and lines represent the median of the group. Statistical analyses were performed using the Mann-Whitney test (*p<0.05, **p<0.01).

Comparison of *L*. *braziliensis*-infection of neutrophils from CL patients *versus* healthy subjects showed that neutrophils from both groups were similarly infected (Fig 1), according to both the percent infection (panel A) and the parasite load (panel B) at all-time points evaluated. No difference was observed between parasite loads in neutrophils from CL patients *versus* healthy subjects: 187[131–249], 227[151–271], 235[126–321] *versus* 205[79–224], 201[136–270], 203[170–289] parasites/100 neutrophils, after 10, 90 and 180 minutes respectively. Despite this, the number of parasites per neutrophils from CL patients increased over time (panel B). These data suggest that *L*. *braziliensis* is taken up by both CL and HS neutrophils at similar rates.

### Expression of activation markers CD66b and CD62L

We evaluated surface expression of neutrophil activation markers to investigate whether infection with *L*. *braziliensis* stimulates neutrophils to assume an activated phenotype. Activation markers were CD62L, an integrin shed from neutrophil surfaces upon activation, and CD66b, a granule marker that increases upon neutrophil degranulation. These surface markers were measured on both *Leishmania*-infected and uninfected “bystander” neutrophils. As the abundance of surface activation markers on CL *versus* healthy subject PMNs was assessed on different flow cytometers, these results are shown in different graphs. Thus the patterns of surface expression can be compared, but the absolute values of fluorescence intensity cannot.

**Fig 2:**
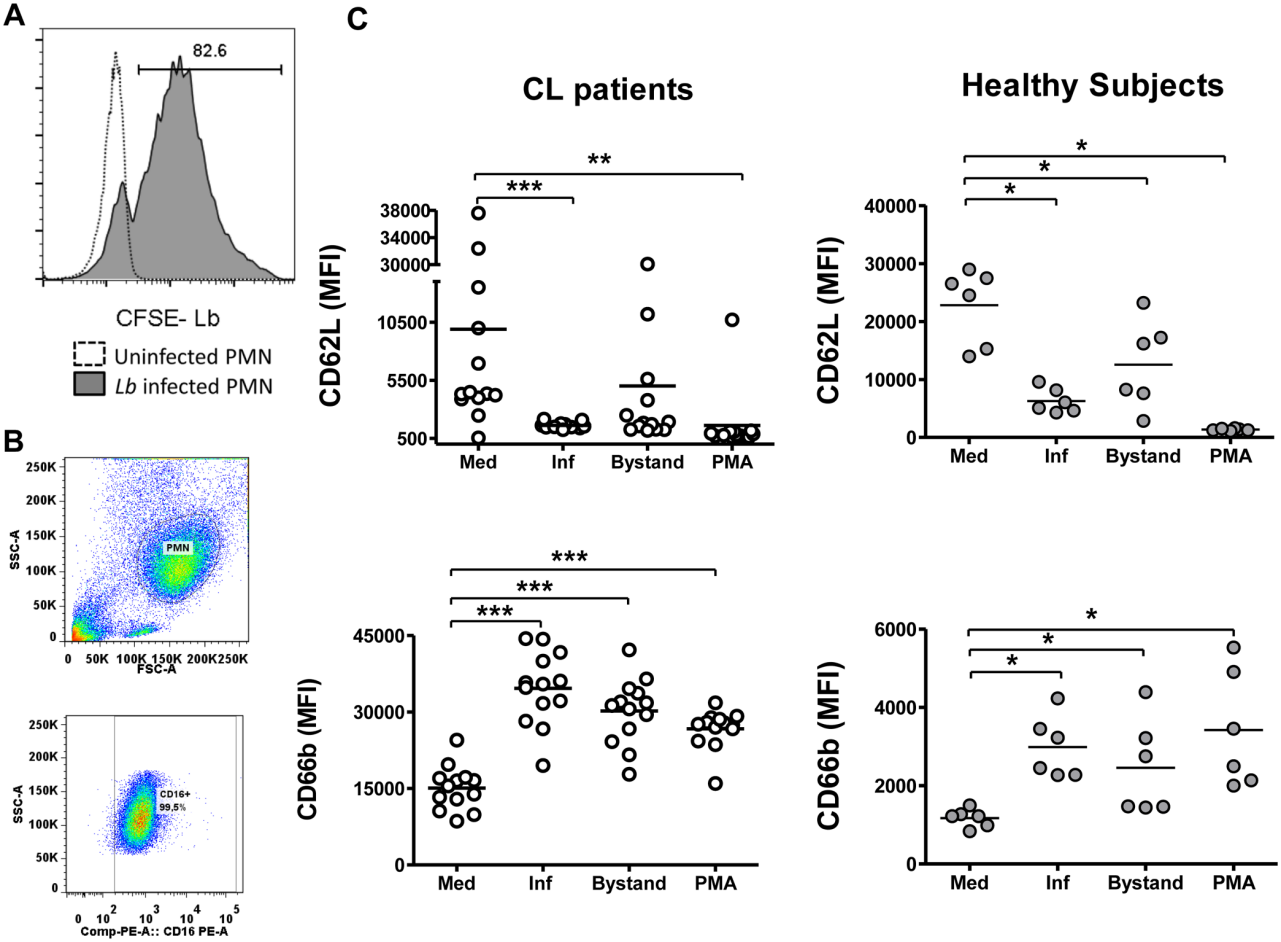
Effect of *L*. *braziliensis* infection on expression of neutrophil activation markers CD62L and CD66b. Panel (A) shows a histogram demonstrating staining in neutrophils infected with CFSE-stained *L*. *braziliensis* versus uninfected neutrophils from a CL patient. Panel (B) shows a representative scatter plot indicating the purity of PMN population based CD16+ expression. Panel (C) shows collated results of surface staining for activation markers CD62L and CD66b by flow cytometry. Each value represents the MFI of one subjects’ neutrophils. “Inf” and “Bystand” represent the staining on neutrophils that were either infected (CFSE+) with CFSE- labeled *L*. *braziliensis*, or uninfected bystander neutrophils (CFSE-). The two left graphs represent data from subjects with CL; the two graphs on the right show data from healthy control subjects. Statistical analysis was performed using the Wilcoxon test, comparing stimulated to unstimulated cells (*p<0.05, **p<0.01, ***p<0.001).

Flow cytometry was sufficient to detect CFSE-labeled *L*. *braziliensis* in infected and uninfected populations of neutrophils (Fig 2A). The surface *L*. *braziliensis* infection led to a significant reduction in surface CD62L on infected neutrophils (CFSE+) compared to basal state, unexposed (CFSE-) neutrophils (Fig 2C). A similar reduction was also observed on neutrophils stimulated with PMA. Additionally, CD62L decreased significantly on the surface of uninfected bystander neutrophils from healthy subjects, although the decrease did not reach statistical significance in subjects with CL. During this short incubation time (90 minutes) it seems likely that any internalized parasites would remain morphologically intact even if killed by phagocytosis (S2 Fig). Thus it seems likely that bystander cells were likely truly uninfected.

Surface CD66b on neutrophils from CL subjects also increased significantly on neutrophils from CL or healthy subjects after incubation with *L*. *braziliensis* or with PMA (Fig 2C). Similar to above CD62L results, changes were observed both in infected and bystander neutrophils, although at to a lower magnitude in bystander cells. Together, these data suggest that *L*. *braziliensis* or PMA can trigger neutrophil activation. Furthermore, these responses did not differ between subjects with active CL or healthy control subjects.

### Neutrophils from CL patients produce higher levels of reactive oxidants than neutrophils from healthy subjects after *L*. *braziliensis* infection

**Fig 3:**
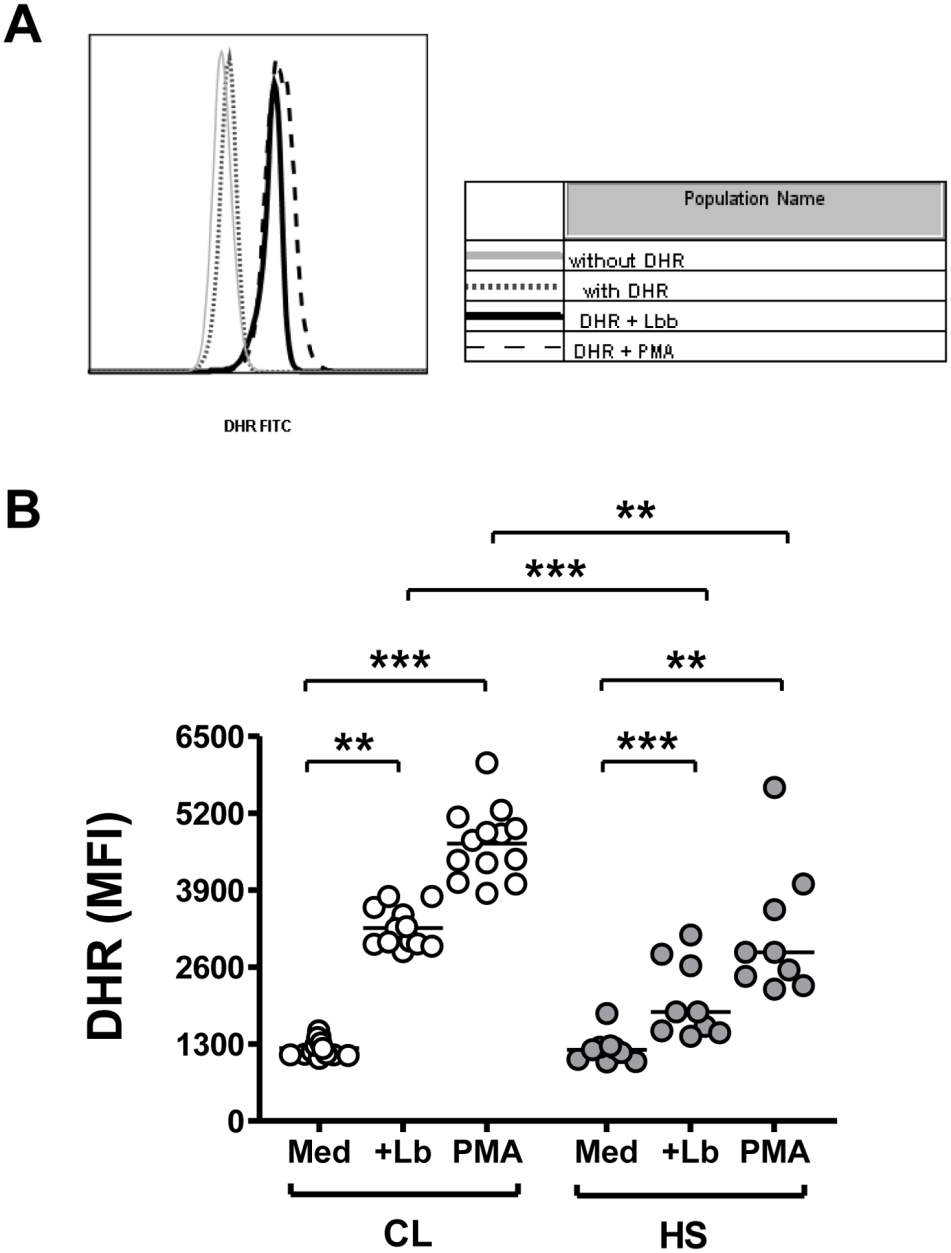
Release of reactive oxidants by neutrophils from subjects with CL or healthy control subjects (HS) induced by phagocytosis of *L*. *braziliensis*. (A) Representative histograms showing DHR-123 fluorescence due to reactive oxidants in unexposed neutrophils from a healthy subject, or the same neutrophils exposed to either *L*. *braziliensis* or PMA. (B) Graphical presentation of the MFI of DHR-123 staining in neutrophils from subjects with CL (n = 13) or healthy control subjects (n = 9), incubated with medium, *L*. *braziliensis* or PMA. Horizontal lines indicate the mean MFI of all subjects. Statistical analyses were performed using the Mann-Whitney test to evaluate differences between the groups, and the Wilcoxon test to compare results of different conditions in the same subject. (**p<0.01, ***p<0.001).

As neutrophils from both CL patients and controls presented a similar phenotype, the functional profiles of these cells were investigated. We first evaluated the capacity of *Leishmania* parasites to trigger oxidant production in neutrophils. Fluorescence of DHR-123, an indicator of the abundance of cellular reactive oxidants, was measured by flow cytometry (Fig 3 panel A).

Following exposure to either *L*. *braziliensis* or to PMA, neutrophils from both groups of subjects released significantly greater amounts of oxidants than neutrophils incubated in basal conditions (Fig 3B). However, the abundance of reactive oxidants produced by neutrophils from CL patients was significantly greater than that generated by healthy controls in response to either stimulus (Fig 3B). Thus, despite similar levels of infection (Fig 1), these data suggest that neutrophils form CL patients are capable of producing significantly more reactive oxidants than neutrophils from healthy controls after *in vitro* exposure to *L*. *braziliensis*.

### Higher amounts of reactive oxidants are not associated with control of parasites

To investigate whether high levels of reactive oxidants produced by neutrophils from CL patients could control the growth of intracellular parasites, NADPH oxidase was inhibited with DPI and the number of infected cells and the parasite burden were evaluated. The data showed there was no difference between the frequency of infected neutrophils or the total parasite burdens in neutrophils incubated in the presence or absence of DPI (89.6±5.4 *versus* 82±6.3 percent infected, respectively), or (242.2±48 *versus* 187.6±34 parasites per 100 neutrophils, respectively). These data suggest that high levels of ROS produced by neutrophils from CL patients do not result in parasite killing.

In order to confirm that neutrophils did not participate in the parasite killing we assessed parasite viability. There was no difference between the number of live promastigotes recovered from *L*. *braziliensis* infected neutrophils from CL patients compared to those from healthy subjects after 24 hours of culture (5.3x 10^6^ parasites/ml ±1.8 *versus* 5x10^6^ parasites/ml ±0.7). This demonstrates that, in this time frame, neutrophils did not participate in the control of intracellular *L*. *braziliensis* proliferation.

### Production of chemokines in response to *L*. *braziliensis* infection of neutrophils

**Fig 4:**
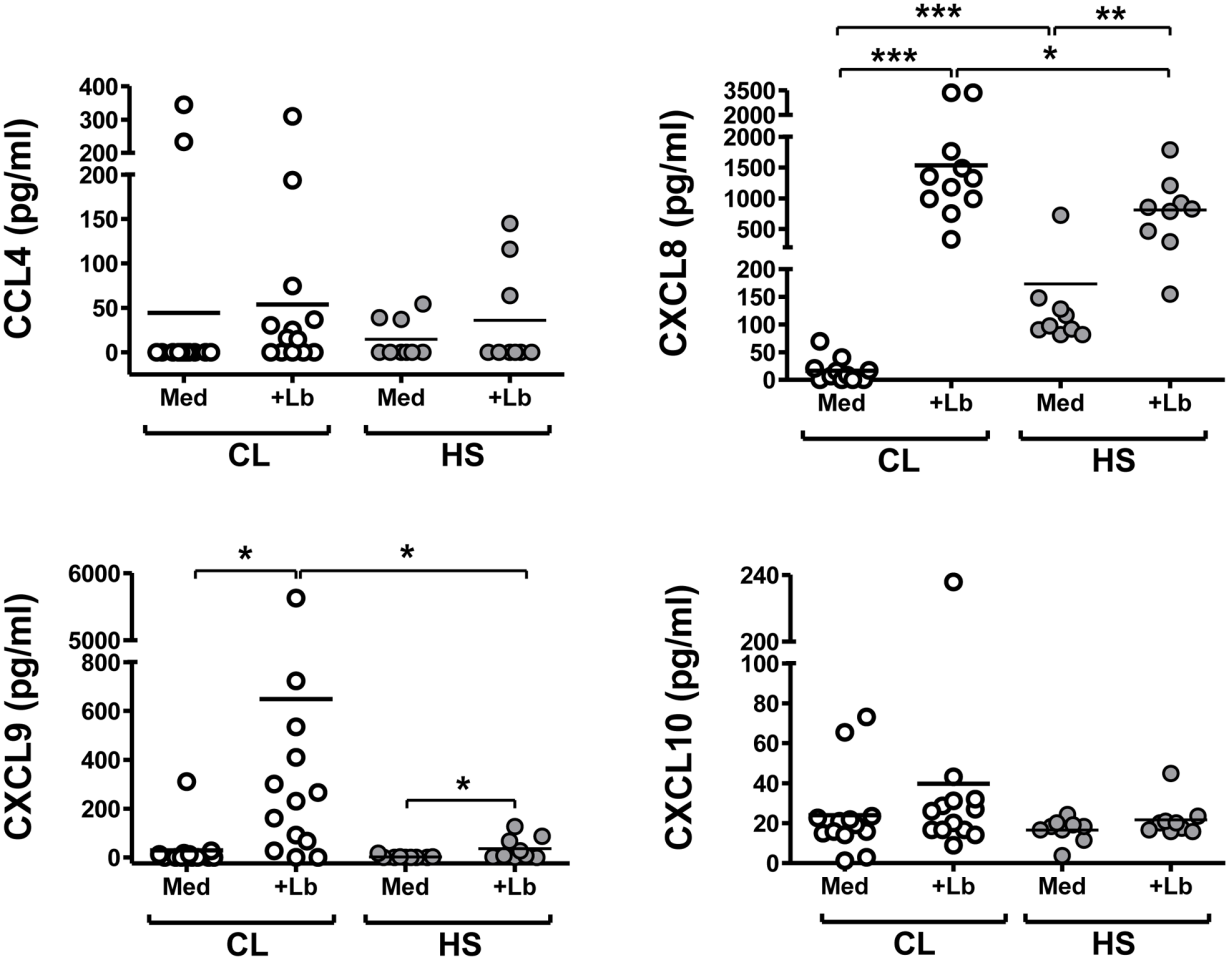
Chemokines produced by neutrophils from CL patients or healthy controls. Neutrophils from CL patients (n = 13) or healthy controls (n = 9) were incubated with *L*. *braziliensis* at a 5:1 ratio for 180 minutes. The concentrations of the indicated chemokines in the supernatants were evaluated by ELISA. Each symbol represents mean values from a different patient, and lines represent the median of each group. The Mann-Whitney test was used to compare differences between CL *versus* Healthy Control groups (*p<0.05, ***p<0.001).

The production of chemokines was measured as an additional measure of neutrophils function. Thus CCL4, CXCL8, CXCL9 and CXCL10 were measured in supernatants from unstimulated or *L*. *braziliensis*-infected neutrophils from CL patients or healthy subjects using ELISA (Fig 4).

The basal production of CXCL8 was significantly lower in CL patient neutrophils than neutrophils from healthy subjects (P<0.001). *Leishmania braziliensis* exposure significantly enhanced CXCL8 production by both groups, although the level reached a higher average in CL subjects than healthy controls (Fig 4). Additionally, the production of CXCL9 by neutrophils from CL *versus* healthy subjects induced by *L*. *braziliensis* exposure was significantly higher than neutrophils from healthy subjects. The abundance of CCL4 was low or below the assay detection level in samples from most subjects, and there was no difference in CXCL10 production between neutrophils from CL compared to healthy subjects.

### Changes in neutrophil profiles after treatment of cutaneous leishmaniasis

**Fig 5:**
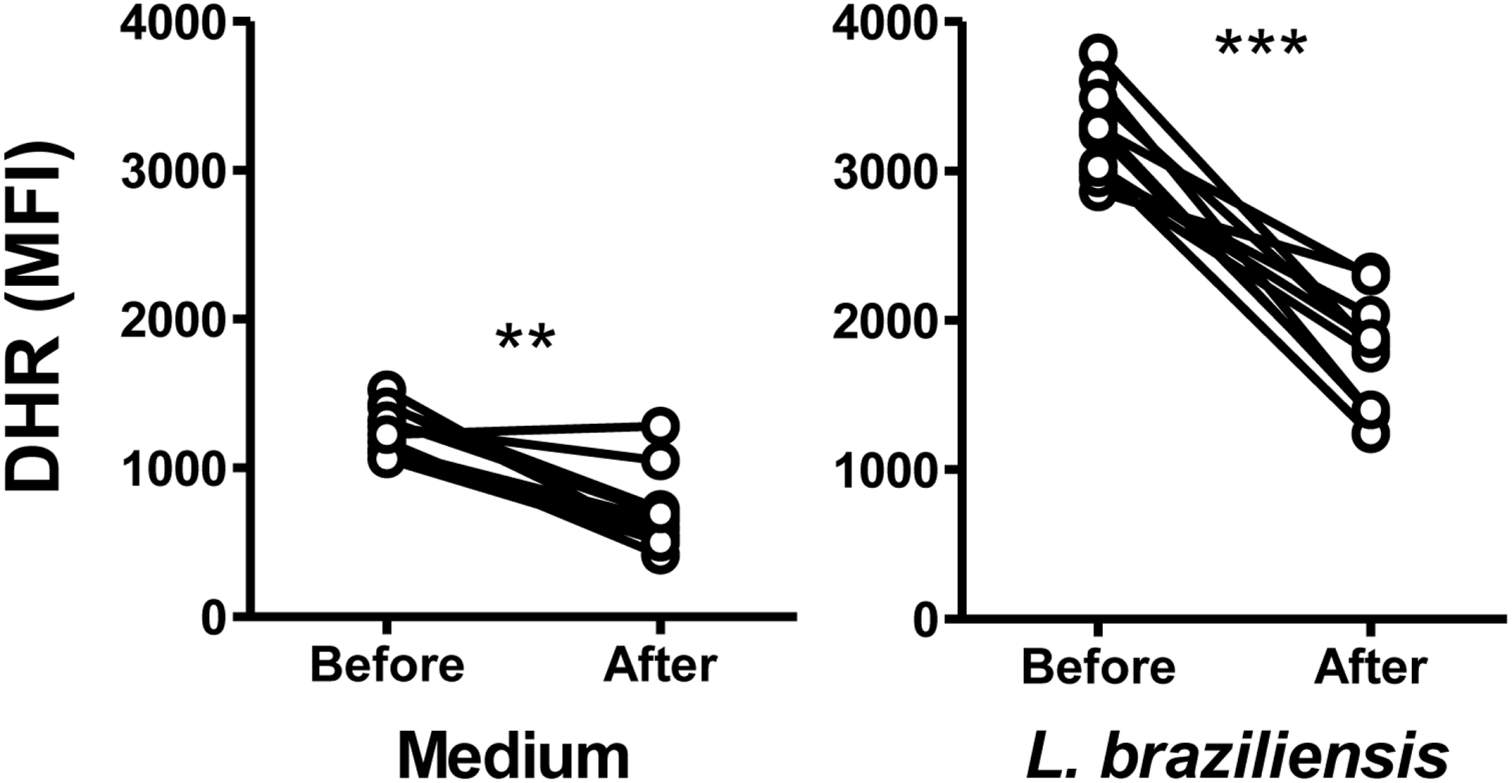
Neutrophil reactive oxidant production from subjects with CL before or after treatment. Neutrophils from CL patients (n = 11) were obtained before and after successful treatment of the disease, in which there was local resolution of the lesion. Cells were incubated in dihydrorhodamine 123 (DHR-123), and incubated with medium alone, 5:1 *L*. *braziliensis*, or PMA for ten minutes. The concentration of reactive oxidant species in neutrophils was assessed by flow cytometry. Data represent the median MFI samples from each patient. Statistical analyses were performed using the Wilcoxon test. (**p<0.01, ***p<0.001).

**Fig 6:**
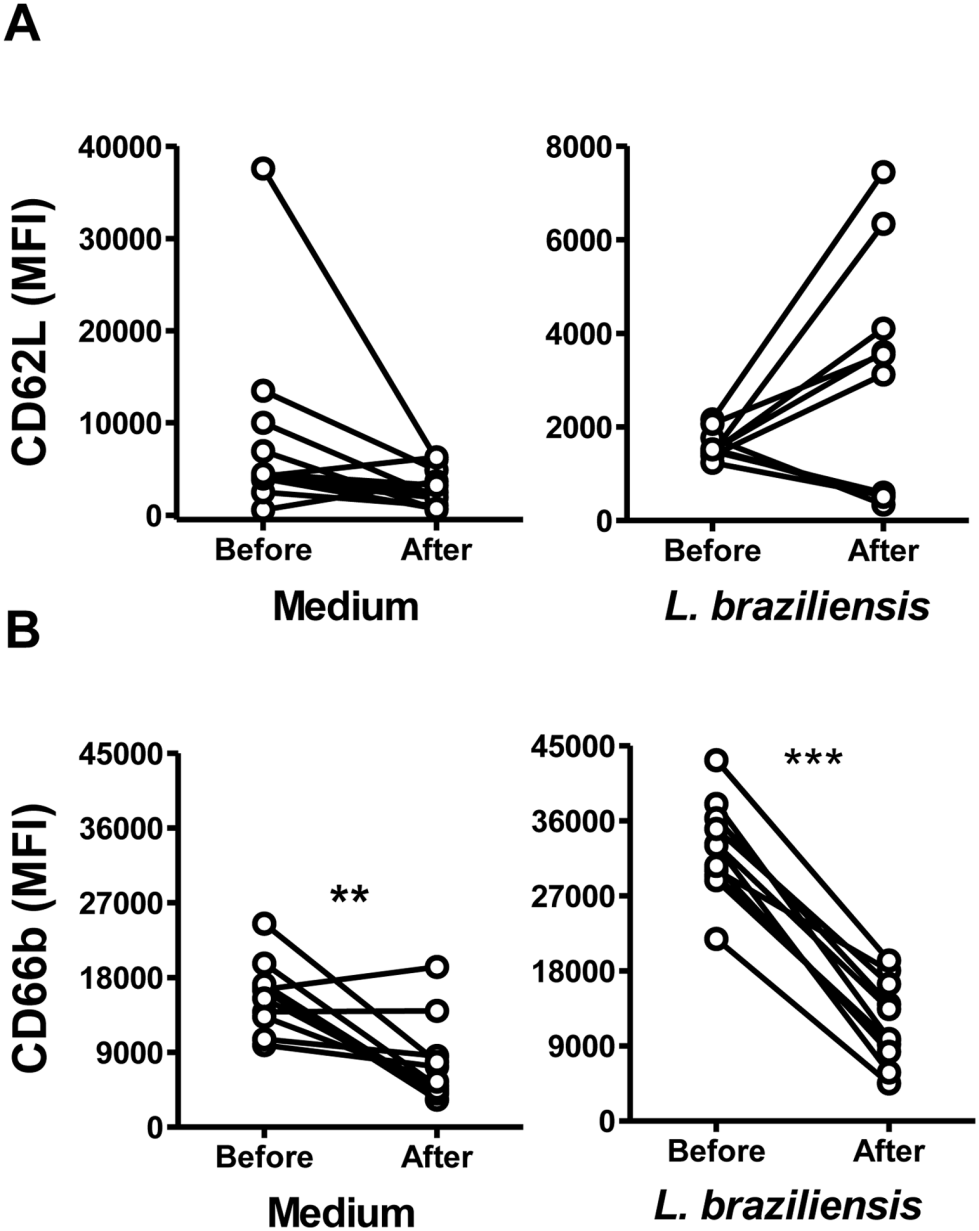
Expression of neutrophil surface markers CD62L and CD66b from CL patients before or after therapeutic cure. Neutrophils from CL patients (n = 11) were isolated before or after CL treatment, and incubated with CFSE stained *L*. *braziliensis* at a 5:1 ratio. After 90 minutes cells were stained for flow cytometry and the expression of CD62L (A) and CD66b (B) was assessed by flow cytometry. Points on graphs represent the median MFI for neutrophils from each subject, incubated in medium alone or with CFSE labeled *L*. *braziliensis*. Statistical analyses utilized Wilcoxon test (**p<0.01, ***p<0.001).

To evaluate whether neutrophil characteristics were modified by successful treatment of patients with leishmaniasis, we compared the oxidative responses of neutrophils from the same CL patients before therapy, and after treatment-induced cure. The abundance of both the spontaneously produced reactive oxidants, and oxidants produced in neutrophils incubated with *L*. *braziliensis*, were significantly lower in subjects after treatment than before (Fig 5). There was no difference observed in CD62L expression on neutrophils from CL patients before and after cure (Fig 6A). However, CD66b expression was significantly lower on both unstimulated (medium) and *L*. *braziliensis*-stimulated neutrophils from subjects after cure (Fig 6B).

**Fig 7:**
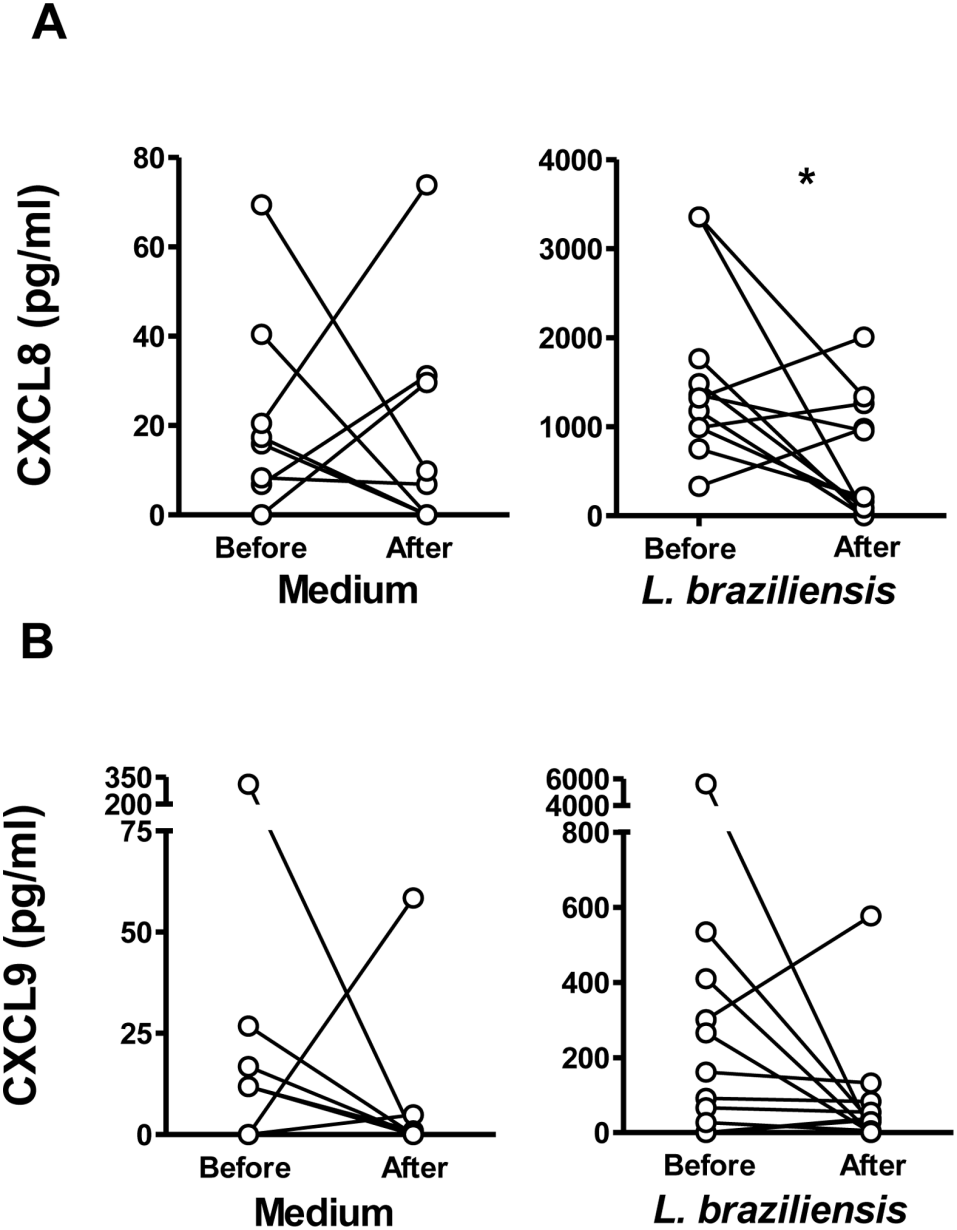
Chemokine production by neutrophils from patients with CL before or after treatment. Neutrophils from CL patients (n = 11) were obtained before or after therapeutic cure, and incubated with or without 5:1 *L*. braziliensis for 180 minutes. The release of of CXCL9 (A) or CXCL8 (B) into supernatants was evaluated by ELISA. Data points show the median of triplicate samples for each subject/condition. Statistical analyses were performed using Wilcoxon test (*p<0.05).

A comparison of CXCL8 and CXCL9 released by neutrophils from CL patients before or after successful treatment is shown in Fig 7. CXCL8 produced by *L*. *braziliensis*-exposed neutrophils from CL subjects after treatment was significantly lower than before treatment (Fig 7A). Analysis revealed no significant differences between CXCL9 levels before or after treatment (Fig 7B), although careful examination of the data revealed all but two subjects exhibited a drop in CXCL9.

## Discussion

Studies of the pathogenesis of human CL have revealed a fine balance between type 1 adaptive immune responses leading to parasite clearance and exaggerated inflammatory responses leading to tissue damage [2,5]. As an illustration, IFN-γ and TNF, which are required for cure of infection in mouse models, do not prevent ulceration in humans and actually correlate with the development of disease [5]. Levels of IFN-γ and TNF directly correlate with lesion size [5] and the levels fall after successful therapy [4]. Moreover, immunomodulators that downmodulate the immune response and decrease TNF production, such as GM-CSF or pentoxyfilline, are more effective than antimony alone at reducing the time to healing and promoting cure of patients who are refractory to treatment with antimony alone [36,37]. Furthermore, peripheral blood cells from individuals with subclinical infection, detected by a positive delayed type hypersensitivity test (DTH) to soluble leishmanial antigen (SLA) with no history of symptomatic disease, produce lower levels of these cytokines than CL patients [6].

Although neutrophils have been observed in CL lesions [38], a role for these cells in the pathogenesis of *L*. *braziliensis* disease pathogenesis has not been defined. Neutrophils are generally thought to be short-lived hematopoietic cells that migrate quickly to sites of infection. In mice, neutrophils migrate in large numbers to tissues infected with *L*.*braziliensis* [21,39]. Neutrophils are also found in tissues of CL patients albeit usually in small numbers [40,41]. In contrast, macrophages and lymphocytes are the main hematopoietic cells at the site of inflammation in patients with CL, after several weeks to months of infection when biopsies are usually performed [38,42]. The current study was based on the hypothesis that neutrophils contribute to the inflammation observed in human CL. Neutrophils can migrate to the site of infection and may produce inflammatory mediators in response to *L*. *braziliensis* infection triggering adaptive immune response, and thus could have an impact on the outcome of disease.

Our data show that the frequency of infected cells in neutrophils from both CL patients and healthy subjects remained unchanged over a 180 minutes course of *in vitro* infection, showing that neutrophils from both groups were similarly susceptible to *L*. *braziliensis* infection. However, we observed increased parasite loads in CL patient neutrophils during increased lengths of parasite exposure. Previous studies have been demonstrated that blocking neutrophil CR3 reduces the uptake of *L*. *braziliensis* [23] and TLR2 expression increases after *L*. *braziliensis* infection [24]. It is possible that neutrophils from CL patients may increase their expression of these receptors associated with parasite uptake, and this may influences parasite burden.

Following infection, neutrophils from both CL patients and healthy subjects presented a similar pattern of activation characterized by increased CD66b and decreased CD62L expression. CD66b is endogenous in specific granules and its increased appearance on the PMN surface indicates exocytosis from specific granules [43]. CD62L, also called L-selectin, is a homing receptor that is cleaved from the neutrophil surface upon activation, and its loss facilitates migration out of the circulation [44]. The combined changes in both surface markers is indicative of activated phenotype [23,45]. Similarly, activated neutrophils were observed in a murine model of *L*. *braziliensis* infection [21] and studies of *L*. *amazonensis*-infected human neutrophils [24] showed that neutrophils from patients with CL due to a different organism a decrease in CD62L after exposure to the parasite. We also observed that, like infected cells, bystander neutrophils also presented an activated phenotype. This could have occurred due to exposure to infected neutrophils, and/or to transient contact with parasites. Alternatively, it has been demonstrated that exosomes, released from *Leishmania* spp. parasites have proinflammatory properties [46] and can activate resting neutrophils [47] or dendritic cells [48]. Furthermore, bystander dendritic cells express high levels of class II, CD80 and CD86 after exposure to *L*. *braziliensis*, and their activation has been shown to require both, and host TNF [48].

Innate anti-microbial mechanisms of neutrophils include generation of reactive oxygen species (ROS), release of granule contents [14], and production of neutrophil extracellular traps (NETs) [49,50]. Phagocytosis can activate neutrophil NADPH oxidase, generating reactive oxygen species that can contribute to the elimination of internalized microorganisms [23]. Data shown in the current report document an increase in ROS generation upon *L*. *braziliensis* infection of neutrophils from CL patients compared to controls. This result agrees with studies showing that *L*. *braziliensis* triggers ROS production by murine neutrophils [21,22]. Monocytes from patients with CL also produce ROS after exposure to *L*. *braziliensis*, and in this cell the ROS may contribute to control of parasite replication [51,52]. In contrast to monocytes, we did not detect evidence that the excess ROS generated by infected CL neutrophils contributed to control of intracellular parasite level. As further evidence for its lack of effect, inhibiting ROS generation by inhibition of the NADPH oxidase in neutrophils did not alter either the number of infected cells or the number of internalized parasites.

Recently, roles for neutrophils in the pathogenesis of leishmaniasis have been explored both *in vitro* and using mouse models. Phagocytosis of apoptotic leishmania-infected neutrophils by macrophages results in transfer of live parasites to macrophages, while changing the macrophage phenotype to an anti-inflammatory state characterized by production of TGF-β [53]. This has raised the hypotheses that neutrophils harboring intracellular leishmania may act as a “Trojan Horse”, serving to both pass live parasites to macrophages and inhibit macrophage microbicidal activity. Neutrophils have opposing effects in vivo depending on the genetic background of the host mouse [54]. Thus, neutrophil depletion from genetically susceptible BALB/c mice infected with *L*. *major* decreased parasite burden, whereas neutrophil depletion did not affect the development of a protective type 1 response in genetically resistant C57BL/6 mice [15]. Similarly, neutrophil depletion from BALB/c mice infected with *L*. *amazonensis* increased both parasite burden and lesion size, whereas neutrophil depletion did not modify the course of *L*. *amazonensis* infection in resistant C57BL/6 mice [16]. This may be in part due to phenotypic differences between neutrophils from susceptible and resistance mice; BALB/c neutrophils express lower levels of TLR2, TLR7 and TLR9 and secrete lower amounts of IL-12p70 after *L*. *major* infection than C57BL/6 neutrophils [17]. Both results suggest either a protective or an indifferent role for neutrophils in disease pathogenesis. Our data suggest neutrophils may be indifferent to control of parasite loads, but might contribute to the inflammatory state of the host.

The above reports in murine models of leishmaniasis raise the hypothesis that infiltrating neutrophils may influence the development of adaptive immune responses to *L*. *braziliensis* in humans. Although we cannot directly test this hypothesis, the release of chemokines and cytokines from infected neutrophils suggests they may influence cellular responses. Other reports have documented neutrophils producing chemokines and cytokines including CXCL8, CXCL9, CXCL10, IFN-γ, IL-12, CCL3, CCL4, IL-17 and IL-23 [55,56]. Of particular interest to us in this study were CXCL8 which induces neutrophil migration, and CXCL9 and CXCL10 which participate in recruitment of Th1-type lymphocytes [2,5]. Also of interest is CCL4, which recruits monocytes and NK among others [57]. Although CCL4 and CXCL10 levels did not differ between CL and healthy control neutrophils, the chemokines CXCL8 and CXCL9 were augmented in neutrophil supernatants from subjects with CL. These chemokines may participate in the recruitment of neutrophils and T cells to the site of *L*. *braziliensis* infection, thus contributing to the overall inflammatory state. Our findings do not suggest that the previous report of *L*. *major* inhibition of neutrophil CXCL10 can be to generalized to *L*. *braziliensis* [58], but our data do suggest that neutrophils augment CXCL9 similar to the reported increase in macrophages from CL patients [59]. It remains to be seen whether differences or similarities can be attributed to different host responses of human neutrophils to distinct *Leishmania* species.

Our data show that circulating peripheral blood neutrophils from patients with CL were more activated, they produced higher levels of reactive oxidants and they generated higher amounts of the proinflammatory chemokines CXCL8 and CXCL9 than neutrophils from healthy subjects. These neutrophil changes were largely reversed after successful therapy of CL. Surprisingly, the heightened activation state and greater ROS production by neutrophils from CL subjects did not result in a greater capacity to control intracellular parasites. These data suggest that neutrophils contribute to the inflammatory environment observed in cutaneous leishmaniasis, primarily through the production of inflammatory mediators responsible for the recruitment of T cells and by ROS production, but that they may not contribute to parasite clearance.

Although CL is a localized disease, it is well known that proinflammatory cytokines are increased in plasma [60] and they are generated by peripheral blood lymphocytes stimulation with *Leishmania* antigen [61]. Neutrophils are short lived cells, they can become primed or activated by cytokines produced by T cells including TNF and IFN-γ, resulting in enhanced ROS and chemokine release [62,63]. Thus, circulating CL neutrophils behaved more like primed neutrophils, poised for rapid activation, than resting neutrophils. The reversal of the neutrophil function after therapeutic cure of localized CL supports this hypothesis.

The dissociation between the inflammatory profile and the ability of neutrophils to kill intracellular *Leishmania* killing has been shown in monocytes and macrophages infected with *L*. *braziliensis* [51,59]. This lack of microbicidal activity differed from healthy control neutrophils, suggesting that the circulating inflammatory neutrophil phenotype does not help to clear infection. Additional studies will be required to determine whether this altered circulating neutrophil phenotype is responsible for maintenance of the inflammatory response observed in tegumentary leishmaniasis due to *L*. *braziliensis*. These observations highlight the importance of correlating phenotypic changes with function in circulating and local tissue cellular responses, in order to understand the extent of inflammatory dysregulation that occurs during tegumentary leishmaniasis and other chronic infections.

## Supporting Information

S1 Fig Representative plots used to characterize infected and bystander neutrophils based on side and forward scatter (A) and CFSE expression (B). (TIF)

S2 Fig Microscopic evaluation of *L*. *braziliensis*-infected neutrophils after 90 minutes of parasite exposure. Internalized parasites are indicated by red arrows.
(TIF)
