# Supplementary figures and images for: Characterization of Neutrophil Function in Human Cutaneous Leishmaniasis Caused by Leishmania braziliensis
Source: PLoS Negl Trop Dis. 2016 May 11;10(5):e0004715. doi: 10.1371/journal.pntd.0004715 (PMC4864077; doi:10.1371/journal.pntd.0004715)

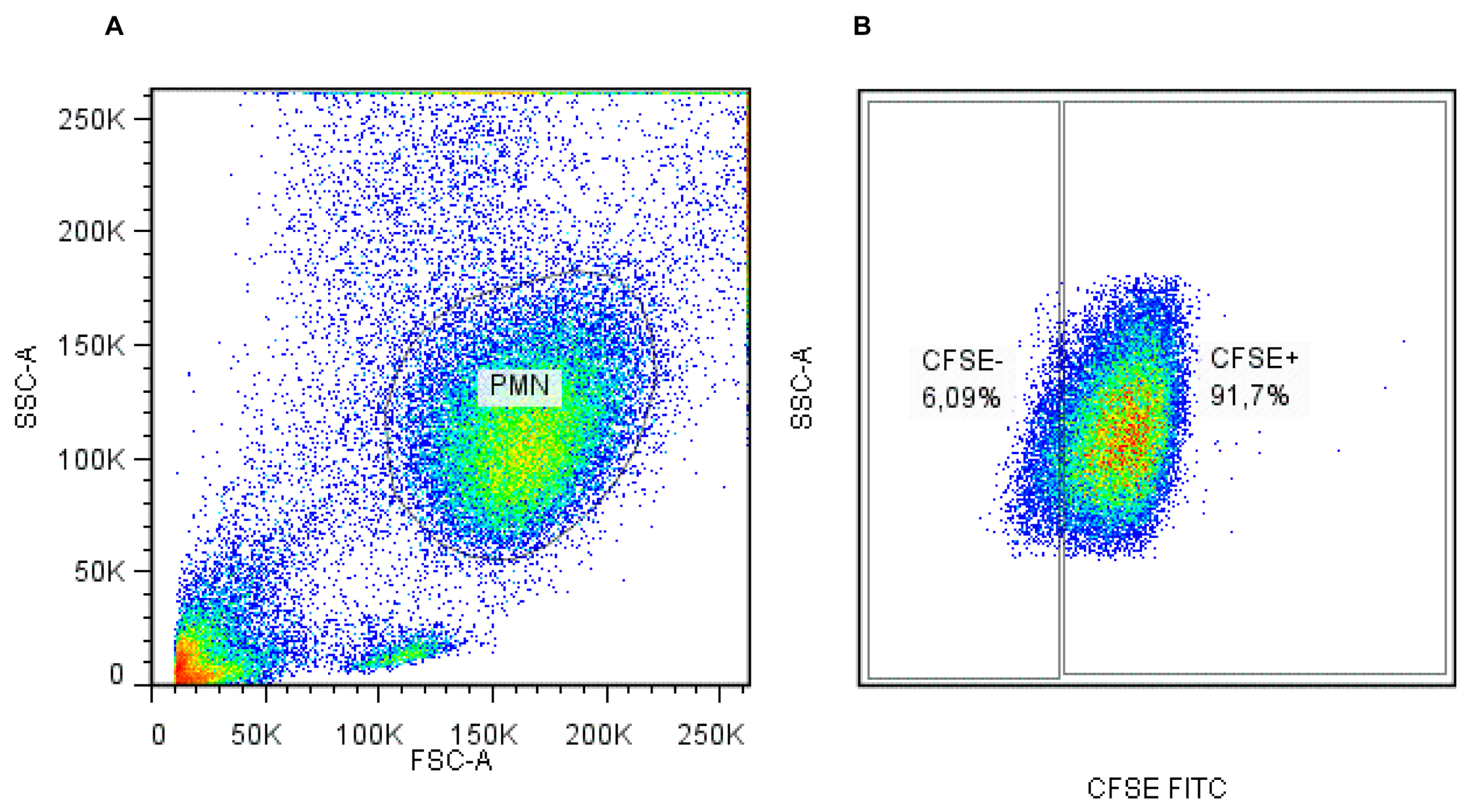

Supplement: S1 Fig — (TIF) [file pntd.0004715.s001.tif]

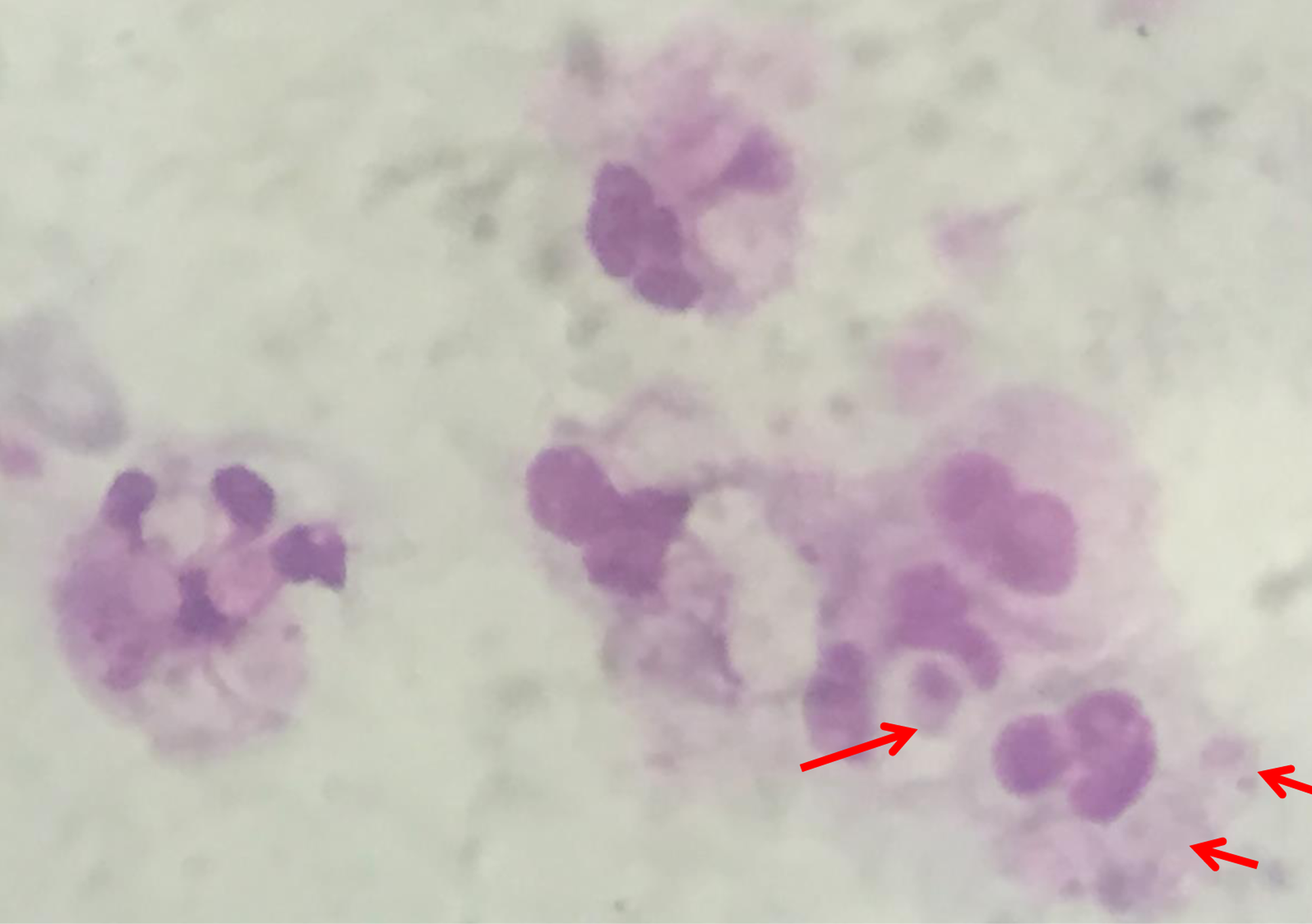

Supplement: S2 Fig — Internalized parasites are indicated by red arrows. (TIF) [file pntd.0004715.s002.tif]
